# Nutritional quality of the food choices of Canadian children

**DOI:** 10.1186/s40795-021-00422-6

**Published:** 2021-05-29

**Authors:** Salma Hack, Mahsa Jessri, Mary R. L’Abbé

**Affiliations:** 1 Department of Nutritional Sciences, Temerty Faculty of Medicine, University of Toronto, Toronto, M5S 1A8 Canada; 2 Food Nutrition and Health Program, The University of British Columbia, Vancouver, V6T 1Z4 Canada

**Keywords:** The Canadian Community Health Survey, Nutrition 2015, Health Canada’s Surveillance Tool, Tier System, Canada’s food guide, Dietary intakes, Diet quality, Nutrition policy

## Abstract

**Background:**

The release of the Canadian Community Health Survey (CCHS), Nutrition 2015 provides a unique opportunity since CCHS 2004 to investigate food choices of Canadian children and adolescents at a national level.

**Objective:**

This study examined the quality and quantity of food choices of children ages 2–8 years and adolescents 9–18 years, using Health Canada’s Surveillance Tool Tier System 2014. It is hypothesized that Canadian children and adolescents are consuming diets poor in nutritional quality based on evidence from the last national nutrition survey in 2004.

**Design:**

Intakes from CCHS 2015, 24-h dietary recall were categorized into Health Canada’s Tiers 1–4, based on CNF/CFG classification system and thresholds for nutrients to limit i.e., total fat, saturated fats, sugars, and sodium to assess quality of food choices. Additionally, dietary intakes were grouped according to Canada’s 2007 food guide servings as the 2019 food guide was not available.

**Results:**

Majority of foods reported by children 2–18 years were categorized as Tier 2 and Tier 3 foods. Investigation of energy contributions from the Tier 4 and “other foods” represented 21–25% of daily calorie intake and of these foods, high fat and/or high sugar foods contributed majority of daily calories to these categories.

**Conclusions:**

This study showed Canadian children 2–18 years are consuming diets high in nutrients recommended to limit. Evidence from this study provides a unique opportunity to improve the nutritional quality of foods, and the food choices of children.

## Introduction

Adequate nutrition and a healthy diet throughout childhood is critical to minimize risks for the development of non-communicable diseases (NCDs) such as, obesity, type- 2 diabetes, and cardiovascular diseases [1, 2]. However, for many Canadians, making the healthy choice has become difficult. Canada’s food environment has changed over the last decade where the availability of convenient and processed foods high in sugar, sodium, total fats, and saturated fats have become increasingly abundant encouraging an obesogenic diet [1, 3, 4]. Within Canada, obesity has been recognized as an epidemic affecting not only adults but children. Children who have unhealthy weights are predisposed to having high blood pressure, increased risk for type-2 diabetes, and additional physical health problems [3]. According to the 2015 Canadian Community Health Survey (CCHS) Nutrition, measured body mass index (BMI) for children ages 5–17 years indicated 19% of children were overweight and 12% of children were obese according to the World Health Organization classification system [4, 5].

To improve harmful dietary behaviours of Canadians, Canada’s Healthy Eating Strategy was released in 2016 [6]. This strategy committed to restricting marketing and advertising of foods poor in nutritional quality to children, improve nutrition labeling, implement mandatory Front-of-Package labelling, and increase nutrition knowledge and awareness [6]. Although implementation of many of these goals are still underway, Health Canada recently released the 2019 Canada’s food guide to help Canadians improve dietary habits [7].

To assess dietary intakes of Canadians, Health Canada developed a nutrient profiling tool i.e., the Health Canada Surveillance Tool Tier system (HCST) in 2014 to evaluate the nutritional quality of foods based on upper and lower thresholds for nutrients of public health concern i.e., sodium, total fat, saturated fats, and sugars [8, 9]. These nutrients have been emphasized to be avoided not only in dietary intakes according to Canada’s new 2019 food guide, but globally by the World Health Organization (WHO) [1, 7]. Recommendations to decrease salt intake by 30% and reduce saturated fat and sugar intake to less than 10% of daily energy have been encouraged globally by the WHO [1, 10].

Current evidence investigating the quality of foods choices available to Canadians and the foods Canadians choose to consume based on the most up-to-date nutrition information available i.e., CCHS 2015 is lacking within Canada. This study aimed to examine the nutritional quality of foods consumed by Canadian children and adolescents 2–18 years according to the HCST nutrient profiling model i.e., foods to choose most often, foods to choose few of, and foods recommended to limit [8, 9]. The results of this study can provide the evidence needed to support recommendations to improve the quality of foods available to children and help inform nutrition policies aimed to reduce the prevalence of NCDs.

## Methods

### Experimental design

The CCHS 2015 is a cross-sectional survey which sampled the Canadian population during January 2, 2015 to December 31, 2015 [11]. The survey design included sampling individuals according to 12 age-sex Dietary Reference Intake (DRI) categories. CCHS 2015 was a voluntary 24-h dietary recall survey conducted using a modified 5 - step automated multi-pass method (AMPM), adapted for the Canadian population, from the United States Department for Agriculture. Respondents completed a 24-h dietary recall, and provided additional information on demographic and lifestyle characteristics. The total sample size of the survey was *n* = 20, 487 individuals with a 61.6% response rate [11]. Following the 24-h dietary recall, 35% of respondents were asked to complete a second day recall over the phone within 3–10 days of the first interview occurring on all days of the week.

### Subjects

Individuals sampled in the survey were ages > 1 year residing in Canada’s 10 provinces, excluding individuals living in: territories, reserves, Aboriginal settlements, full-time members of the Canadian Armed Force, and institutionalized individuals [11]. Results from pregnant and breastfeeding women, infants, and individuals with invalid dietary recalls (as defined by Statistics Canada) were excluded from this study. The nutritional quality of foods consumed from single 24-h dietary recalls for boys and girls between 2 to 18 years (*n* = 4642) were investigated in this study.

### Classifying foods in the 24-h dietary recall according to HCST

Foods reported in the 24-h dietary recall were categorized and the nutritional composition was determined using the 2015 Canadian Nutrient File (CNF) which contained information for 5690 unique Canadian foods commonly consumed [11, 12]. Staff from Health Canada and Public Health Agency of Canada developed the CNF/CFG classification, enabling linkage of CNF food codes to four CFG food groups and 21 subgroups according to Eating well with Canada’s food guide 2007 [12, 13]. Foods in the CCHS 2015 were categorized according to Canada’s Food Guide subgroups, and placed into Tiers according to established thresholds for sodium, saturated fats, total fats, and sugars [8, 9, 13]. These thresholds were derived from thresholds used for nutrient content claims, DRI, and Nutrition Standards for Foods in Schools [8].

Exact methods used by Health Canada to categorize foods into Tier groups are described elsewhere [8, 9]. Briefly, lower thresholds for fats and sodium content were based on nutrient content claims for amounts commonly consumed in one sitting, termed the Reference Amount (RA) [8]. Foods categorized as Tier 1 must not exceed any of the lower thresholds: ≤ 3 g/ RA fat, ≤140 mg/RA sodium and, ≤ 6 g/RA sugar [8]. As there is no daily value (DV) for sugar, upper and lower thresholds for sugar were determined from the recommendation by the Institute of Medicine (IOM) [8]. The 15% DV of sodium (> 360 mg/RA), total fats (> 10 g/RA) and saturated fats (> 2 g/RA) and for sugars (> 19 g) were set as the upper thresholds [8]. Tier 2 foods could exceed one or two lower thresholds but no upper thresholds. Tier 3 foods are foods with nutrient contents above all lower thresholds (i.e., sodium, sugar, and total fat), and may exceed one upper threshold. Tier 4 represents foods that exceed ≥2 upper thresholds, however special consideration was given to foods belonging to the Meat and Alternative, and Milk and Alternative categories as they naturally have higher saturated fat content [8]. Additional adjustments for foods based on directional statements from CFG can be found in HCST [8].

Using the CNF/CFG classification system, 9 groups of foods could not be classified according to Tiers 1–4 [8]. Of these groups, 5 categories of foods were grouped as “other foods” representing foods not recommended in the 2007 Canada’s food guide. These groups are: 1) saturated and/or trans-fats and oils; 2) high-fat and high sugar foods such as, candies, chocolates, and syrups; 3) high calorie beverages ≥40 kcal/100 g; 4) low calorie beverages < 40 kcal/100 g (which exclude water); and 5) alcoholic beverages [8, 13].

### Statistics

Analyses were completed using Statistical Analysis Software (SAS) version 9.4 (SAS Institute Inc., Cary, NC, USA). Bootstrap balanced repeated replication with 500 repeats was used to estimate population parameters i.e., confidence intervals, standard errors, and coefficients of variation. Survey weights provided with the master files were used for all individuals 2 to 18 years, to ensure samples from CCHS 2015 remained nationally representative [11]. Dietary intakes were assessed according to DRI age-sex groupings and adjusted for additional lifestyle measures, which included smoking, physical activity and Body Mass Index (BMI). BMI was determined using measured height and weight, and cut-offs for BMI categorization were derived based on WHO BMI growth curves [11]. PROC SURVEYREG and PROC SURVEYLOGISTIC were used for continuous (e.g., servings from fruit and vegetables) and for categorical (e.g., lifestyle measures) analyses, respectively, adjusting for energy intake, age, and sex where appropriate. Results with two- tailed *p*-value ≤0.05 were reported as statistically significant.

### Identification of implausible reporters

Studies using the CCHS 2015 have recognized a large percentage of under-reporting [14, 15]. Under-reporting occurs most often with many socially undesirable foods or those high in fat and sugars [14–16]. Following previous publications, this study identified individuals as under-reporters, plausible reporters, and over-reporters, based on the comparison of their estimated energy requirement (EER) to total energy expenditure (EER: TEE) [11, 16, 17]. The Institute of Medicine (IOM) developed the EER equation which took into consideration age, sex, BMI and physical activity [18]. For children < 12 years, under-reporters were classified as having a reported energy intake (EI) < 74% of the EER, and over-reporters > 135% of their EER [17]. For children ≥12 years, under-reporters were classified as having an EER less than 70% of what was reported and over-reporters were having an EI over 142% of the EER [16]. If children had no reported physical activity level (PAL) they were categorized as “low active” (< 14 years) and “sedentary” (> 14 years), based on findings by Garriguet et al. [11, 15, 17].

### Approvals

All researchers obtained Reliability Status as outlined in the Policy on Government Security and completed a security check by the Royal Ontario Mounted Police, as required by the *Statistics Canada Act.* Data analyses were completed at the Toronto, Ontario Research Data Centre (RDC) of Statistics Canada in accordance with survey guidelines and procedures. To protect the confidentiality of respondents, RDC Analysts reviewed and released the data presented in this manuscript, to ensure compliance with guidelines developed by Statistics Canada. The data presented in this study was completed exclusively as secondary analyses and all information provided was de-identified and did not require institutional REB approval.

## Results

This study included *n* = 4642 children ≤18 years of age. Investigation of adherence to EWCFG in Table 1, revealed that the intake of Vegetables and Fruits was mainly from Tier 1 foods, with no reported intakes from Tier 4 foods. Children ages 2–3 years, and 4–8 years, were consuming more than the required number of recommended EWCFG servings for all four CFG food categories, with the exception of Vegetables and Fruits for children ages 4–8 years. Children 4–8 years to 14–18 years were not meeting the minimum recommended number of CFG servings for Vegetable and Fruits. For all DRI categories, the majority of CFG servings/day were coming from the Grain Products [mean (SEM)] [5.1(0.2) - 6.8(0.2)], specifically Tier 2 grains [2.5(0.1) – 3.7(0.2)] followed by Vegetables and Fruits [3.8(10.2) - 4.5(0.2)] specifically Tier 1 [2.3(0.2) – 2.9 (0.1)], Milk and Alternatives [1.8(0.1) - 3(0.11)] specifically Tier 3 [0.9(0.1) – 1.7 (0.1)], and Meat and Alternatives [1.5(0.11) - 2.1(0.2)] specifically Tier 3 [0.8(0.1) – 1.2(0.1)]. Furthermore, as age increased, for both boys and girls, the number of food guide servings from Meat and Alternatives increased, whereas with Milk and Alternatives the opposite trend was seen, as older individuals were consuming fewer CFG servings.

**Table 1:** Number of servings per food category presented according to the 2014 Health Canada’s Surveillance Tool (HCST) Tier System nutrient profiling model, among Canadian children 2–18 years

Category	Tier	2–3 Years	4–8 years	9–13 years	9–13 years	14–18 years	14–18 years
				Boys	Girls	Boys	Girls
**Mean (SEM)**							
**Vegetables and Fruits**	Tier 1	2.9 (0.1)	2.8 (0.1)	2.4 (0.2)	2.6 (0.2)	2.3 (0.2)	2.5 (0.2)
	Tier 2	1.1 (0.1)	1.3 (0.1)	1.1 (0.1)	1.1 (0.1)	1.2 (0.1)	0.8 (0.1)
	Tier 3	0.4 (0.1)	0.4 (0.1)	0.4 (0.1)	0.4 (0.1)	0.7 (0.1)	0.4 (0.1)
	Tier 4	0 (0)	0 (0)	0 (0)	0.1 (0)	0 (0)	0 (0)
	Tier 1–3	4.4 (0.2)	4.4 (0.2)	3.9 (0.2)	4.1 (0.2)	4.1 (0.3)	3.8 (0.2)
**Total**	Tiers 1–4	4.5 (0.2)	4.6 (0.2)	4 (0.2)	4.2 (0.2)	4.2 (0.3)	3.8 (0.2)
**EWCFG Rec.**		4	5	6	6	8	7
**Mean (SEM)**							
**Grain Products**	Tier 1	1.2 (0.1)	1.1 (0.1)	1.3 (0.2)	1.4 (0.1)	1.3 (0.2)	1.2 (0.1)
	Tier 2	2.5 (0.1)	3.1 (0.1)	3.5 (0.2)	3 (0.2)	3.7 (0.2)	2.8 (0.1)
	Tier 3	0.8 (0.1)	1.1 (0.1)	1 (0.1)	1 (0.1)	0.8 (0.1)	1 (0.1)
	Tier 4	0.7 (0.1)	0.9 (0.1)	1 (0.1)	1.1 (0.1)	0.6 (0.1)	0.7 (0.1)
	Tier 1–3	4.4 (0.2)	5.3 (0.2)	5.8 (0.2)	5.3 (0.2)	5.8 (0.3)	5.1 (0.2)
**Total**	Tiers 1–4	5.1 (0.2)	6.2 (0.2)	6.8 (0.2)	6.4 (0.2)	6.4 (0.3)	5.7 (0.2)
**EWCFG Rec.**		3	4	6	6	7	6
**Mean (SEM)**							
**Milk and Alternatives**	Tier 1	0.2 (0)	0.2 (0)	0.3 (0)	0.3 (0)	0.2 (0)	0.2 (0)
	Tier 2	1 (0.1)	0.8 (0)	0.6 (0.1)	0.6 (0.1)	0.6 (0.1)	0.4 (0)
	Tier 3	1.7 (0.1)	1.2 (0.1)	1.1 (0.1)	1.2 (0.1)	0.9 (0.1)	1 (0.1)
	Tier 4	0.1 (0)	0.1 (0)	0.1 (0)	0.1 (0)	0 (0)	0.1 (0)
	Tier 1–3	2.9 (0.1)	2.2 (0.1)	2.0 (0.1)	2.1 (0.1)	1.7 (0.1)	1.7 (0.1)
**Total**	Tiers 1–4	3 (0.1)	2.3 (0.1)	2.1 (0.1)	2.1 (0.1)	1.8 (0.1)	1.8 (0.1)
**EWCFG Rec.**		2	2	3–4	3–4	3–4	3–4
**Mean (SEM)**							
**Meat and Alternatives**	Tier 1	0.1 (0)	0.1 (0)	0.1 (0)	0.1 (0)	0 (0)	0.1 (0)
	Tier 2	0.4 (0)	0.3 (0.1)	0.4 (0.1)	0.4 (0.1)	0.6 (0.1)	0.5 (0.1)
	Tier 3	0.8 (0.1)	0.9 (0.1)	0.9 (0.1)	0.8 (0.1)	1.2 (0.1)	1 (0.1)
	Tier 4	0.3 (0.1)	0.3 (0.1)	0.2 (0.1)	0.2 (0.1)	0.2 (0.1)	0.3 (0.1)
	Tier 1–3	1.3 (0.1)	1.4 (0.1)	1.4 (0.1)	1.2 (0.1)	1.8 (0.1)	1.6 (0.1)
**Total**	Tiers 1–4	1.6 (0.1)	1.6 (0.1)	1.6 (0.1)	1.5 (0.1)	2.1 (0.2)	1.9 (0.1)
**EWCFG Rec.**		1	1	1–2	1–2	3	2

Tiers are classified according to lower and upper thresholds for nutrients of public health concern: sodium, saturated fats, total fats and sugars. Lower thresholds: total fat: ≤ 3 g/RA, sugars: ≤ 6 g/RA and sodium: ≤ 140 mg/RA [8]. Upper thresholds: total fat: > 10 g/RA, sugars: > 19 g/RA, sodium: > 360 mg/RA, and saturated fats: > 2 g/RA [8]. Tier 1 foods do not exceed any lower thresholds for total fat, sugar and sodium. Tier 2 may exceed one or two lower thresholds but no upper thresholds. Tier 3 Vegetable and Fruit, and Grain Products: exceeds all lower thresholds and no upper thresholds, or only one upper threshold. Tier 3 Milk and Alternatives, and Meat and Alternatives: exceeds all lower thresholds and no upper thresholds, or only one upper threshold for total fat, sugars, or sodium. Tier 4 Vegetable and Fruit, and Grain Products: exceeds two upper thresholds. Tier 4 Milk and Alternatives, and Meat and Alternatives: exceeds upper thresholds for total fat, sugars, or sodium [8]

Data is presented as mean (SEM: standard error of the mean). Servings per/day are classified according to EWCFG: Eating Well with Canada’s Food Guide. Rec: Recommendations

Results are weighted, nationally representative and energy adjusted

**Fig. 1:**
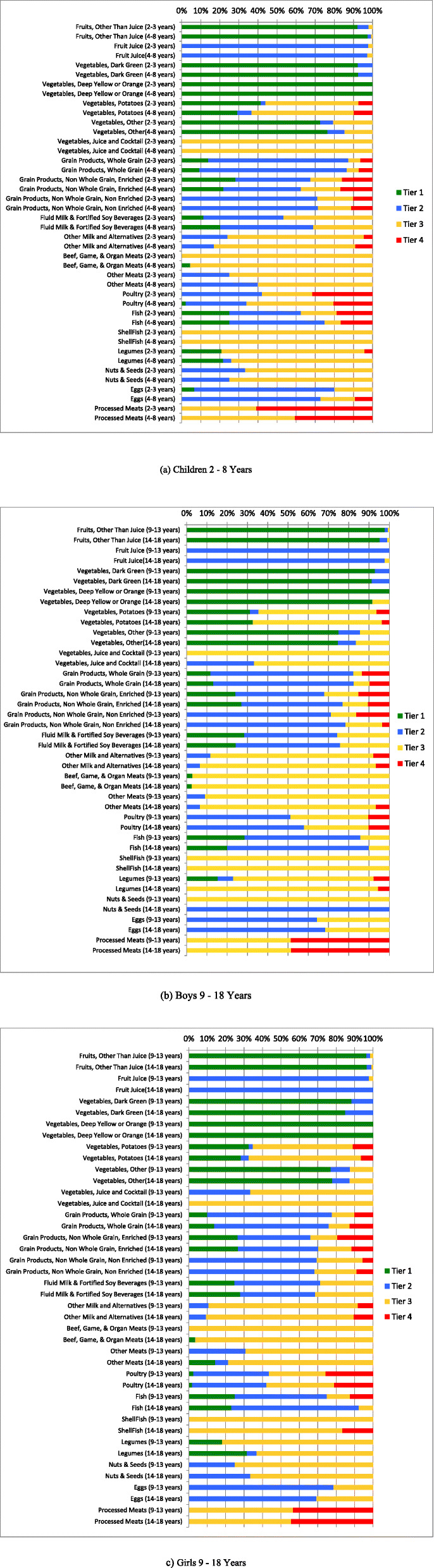
Weighted age-stratified analysis of foods consumed by food category based on the 2014 Health Canada’s Surveillance Tool, Tier system, among children **a** 2–8 years, **b** boys 9–18 years, and **c** girls 9–18 years results are energy adjusted. Tiers are classified according to lower and upper thresholds for nutrients of public health concern: sodium, saturated fats, total fats and sugars. Lower thresholds: total fat: ≤ 3 g/RA, sugars: ≤ 6 g/RA and sodium: ≤ 140 mg/RA [8]. Upper thresholds: total fat: > 10 g/RA, sugars: > 19 g/RA, sodium: > 360 mg/RA, and saturated fats: > 2 g/RA [8]. Tier 1 foods do not exceed lower thresholds for total fat, sugar, and sodium. Tier 2 may exceed one or two lower thresholds but no upper thresholds. Tier 3 Vegetable and Fruit, and Grain Products: exceeds all lower threshold and no upper thresholds, or only one upper threshold. Tier 3 Milk and Alternatives, and Meat and Alternatives: exceeds all lower threshold and no upper thresholds, or only one upper threshold for total fat, sugars, or sodium. Tier 4 Vegetable and Fruit, and Grain Products: exceeds two upper thresholds. Tier 4 Milk and Alternatives, and Meat and Alternatives: exceeds upper thresholds for total fat, sugars, or sodium [8]

Analysis of intakes according to the HCST Tier system (Fig. 1a-c) showed that across all age groups, almost 100% of intakes from Vegetables and Fruits, with the exception of potatoes and vegetable cocktails were from Tier 1 and Tier 2 foods. Intakes of Tier 3 potatoes, which are foods high in either: total fats, saturated fats, sodium, or sugar increased with increasing age groups. Children ages 2–3 years and 4–8 years consumed: 49 and 54% of potatoes categorized as Tier 3 such as some grilled potatoes, or frozen potatoes. Males 9–13 years and 14–18 years consumed: 58, and 64% of potatoes classified as Tier 3, respectively. Females aged 9–13 years, and 14–18 years reported an average intake of 54 and 61% of Tier 3 potatoes, respectively.

Furthermore, HCST classifies the majority of “juice cocktails” as Tier 3 as they exceed one of the upper thresholds; this is reflected in Fig. 1a-c [10]. Across all ages, the majority of intakes from EWCFG Milk and Alternatives, and Meat and Alternatives food categories were foods that CFG recommends to “choose few of” (Tier 3) such as flavoured milks, most cheeses, or deli meats respectively. Within CFG, Milk and Alternatives food group, subcategory “other milk and alternatives”, children 9–13 years-14-18 years reported 80–87% (males), 81–80% (females) of intakes as Tier 3. Examples of foods from this category include processed cheeses or high fat cheeses.

Tier 4 foods are those that are not recommended in EWCFG, and exceed 2 or more of the upper thresholds for sodium, total fat, or sugar. According to Fig. 1a-c, individuals ate more Tier 4 foods belonging to EWCFG categories: Grain Products and Meat and Alternatives, such as certain types of crackers, some cereals, processed meats, or deep fried meat and alternatives. Across all DRI age-sex groups, children ages 2–3 years consumed 61% of processed meats from Tier 4 foods, followed by males 9–13 years and 14–18 years choosing 49 and 48% of Tier 4 processed meats.

Table 2 shows the energy contributions of Tier 4 and “other foods” not recommended by EWCFG by DRI age-group i.e., saturated fats and oils, beverages ≥40 kcal/100 g, beverages < 40 kcal/100 g, high fat/ high sugar foods and alcohol. From 2 to 3 years to 14–18 years, energy contribution from Tier 4 and “other foods” ranged from mean (SEM) [224 (12.8) – 560 (24.8) kcal/day]. Across all DRI categories, energy contribution from “other foods” not recommended by EWCFG, continued to increase, with the largest calorie contribution coming from high fat and/or sugar foods [65(4.7)- 163(16.5) kcal/day]. Overall, energy intake from Tier 4 and “other foods” accounted for 21–25% of the total daily intake for children ages 4–8 years to 14–18 years.

**Table 2:** Weighted analysis of energy contribution from Tier 1–3 and Tier 4 (foods recommended to limit) and “other foods” not recommended by Canada’s Food Guide

	2–3 years	4–8 years	Boys, 9–13 years	Girls, 9–13 years	Boys, 14–18 years	Girls, 14–18 years
Variable (kcal/day)	Mean (SEM)	Mean (SEM)	Mean (SEM)	Mean (SEM)	Mean (SEM)	Mean (SEM)
Tiers 1 + 2 + 3	1094 (26.7)	1232 (22.9)	1443 (26.6)	1304 (25.8)	1760 (45.6)	1228 (26.2)
Tiers 4	119 (10.6)	177 (9.4)	223 (17)	221 (15.8)	214 (17.8)	148 (11.3)
Other Foods	**Other Food/Beverages**					
Alcoholic Beverages	0 (0)	0.6 (0.6)	0.6 (0.3)	0.3 (0.3)	12.4 (4)	4.7 (1.8)
Beverages, High calorie (≥ 40 kcal/100 g)	10 (2.2)	26 (2.6)	58 (5.0)	52 (5.0)	93 (8.3)	68 (6.3)
Beverages, Low calorie (< 40 kcal/100 g)	7 (1.2)	11 (1.9)	19 (2.2)	19 (2.2)	40 (4.7)	21 (2.2)
High fat and/ or sugar foods	65 (4.7)	110 (6.9)	151 (9.0)	140 (10.1)	155 (11.5)	163 (16.5)
Meal replacements	2 (1.5)	3 (1.2)	2 (1.0)	1 (0.5)	2 (1.3)	3 (1.6)
Saturated and/or trans fats and oils	23 (3.1)	31 (2.4)	42 (3.8)	35 (2.8)	46 (4.2)	36 (3.7)
Supplements	1 (0.4)	0.1 (0.1)	1 (0.6)	1 (0.5)	5 (1.6)	5 (1.7)
Uncategorized (ingredients, seasonings, and unprepared foods)	6 (1.9)	6 (1)	6 (0.6)	7 (0.9)	23 (4.5)	9 (1.3)
Unsaturated fats and oils	32 (2.5)	36 (1.9)	52 (3.5)	45 (3.6)	70 (5.5)	52 (3.3)
Total energy from Tier 4 and “Other” foods/beverages (kcal/day)	224 (12.8)	355 (13.3)	493 (21.3)	465 (20.8)	560 (24.8)	441 (26.6)
Total energy from Tier 4 and “Other” foods/beverages (%)	16 (0.8)	21 (0.7)	24 (0.8)	24 (0.9)	23 (0.9)	23 (1.0)

Tiers are classified according to lower and upper thresholds for nutrients of public health concern: sodium, saturated fats, total fats and sugars. Lower thresholds: total fat: ≤ 3 g/RA, sugars: ≤ 6 g/RA and sodium: ≤ 140 mg/RA [8]. Upper thresholds: total fat: > 10 g/RA, sugars: > 19 g/RA, sodium: > 360 mg/RA, and saturated fats: > 2 g/RA [8]. Tier 1 foods do not exceed lower thresholds for total fat, sugar and sodium. Tier 2 may exceed one or two lower thresholds but no upper thresholds. Tier 3 Vegetable and Fruit, and Grain Products: exceeds all lower threshold and no upper thresholds, or only one upper threshold. Tier 3 Milk and Alternatives, and Meat and Alternatives: exceeds all lower threshold and no upper thresholds, or only one upper threshold for total fat, sugars, or sodium. Tier 4 Vegetable and Fruit, and Grain Products: exceeds two upper thresholds. Tier 4 Milk and Alternatives, and Meat and Alternatives: exceeds upper thresholds for total fat, sugars, or sodium [8]. Tier 4 and “other foods” have no Canada’s food guide serving size recommendation. Low calorie beverages (< 40 kcal/100 g) exclude water. Results are presented as Mean (SEM: standard error of the mean)

Table 3 shows the characteristics of the study population grouped into quartiles (Q1-Q4), based on energy intake from Tier 4 and “others foods”. Q1: Compliers < 10.9% energy; Q2 and Q3 intermediates 10.9 - 20.8% energy and 20.9 -32.9% energy, respectively, and Q4: non - compliers > 32.9% energy. There was a small difference between mean ages across all quartiles (Q1-Q4) mean (SEM); [10.1(0.21) - 11(0.21) *p* = 0.0108]. BMI (kg/m^2^) (*p* = 0.9563) and physical activity (*p* = 0.6587) levels remained constant between Q1-Q4. Across Q1-Q4, there was a significant difference between the percentage of under-reporters [32.8 (2) - 19.8 (2)] and over-reporters [10.9 (1) - 19.5 (2); *p* < 0.0001].

**Table 3:** Weighted analysis of characteristics of compliers, intermediates, and non-compliers based on the percentage of energy from Tier 4 foods and “other foods” and beverages among Canadian children 2–18 years

Characteristics	Compliers (Q1)	Intermediates (Q2)	Intermediates (Q3)	Non-compliers (Q4)	p-Trend
	< 10.9% Energy	10.9 -20.8% Energy	20.9 -32.9% Energy	> 32.9% Energy	
	Mean (SEM)	Mean (SEM)	Mean (SEM)	Mean (SEM)	
Age (years)	10.1 (0.21)	10.2 (0.23)	10.7 (0.22)	11.0 (0.21)	0.0108
Sex (%)					
Males	47.4 (2)	50.1 (2)	53 (2)	46.2 (2)	0.9499
Females	52.6 (2)	49.9 (2)	47 (2)	53.8 (2)	
BMI (kg/m^2^)	20 (0.21)	20.1 (0.19)	19.9 (0.20)	20.1 (0.24)	0.9563
Misreporting					
Under-reporter (%)	32.8 (2)	25 (2)	23.3 (2)	19.8 (2)	
Over-reporters (%)	10.9 (1)	15.2 (1)	16.4 (1)	19.5 (2)	<.0001
Physical Activity (%)					
Sedentary	27.3 (2)	28.4 (2)	29.6 (2)	28.2 (2)	0.6587
Low Activity	40.3 (2)	41.6 (3)	43.1 (3)	41.4 (3)	
Very Active	6.6 (1)	6.3 (1)	5.9 (1)	6.3 (1)	

Results are presented as mean (SEM: standard error of the mean) adjusted for age and sex. Quartiles are based on the percentage of energy reported for all Tier 4 foods and “other” foods and beverages classified according to lower and upper thresholds for nutrients of public health concern: sodium, saturated fats, total fats and sugars. Lower thresholds: total fat: ≤ 3 g/RA, sugars: ≤ 6 g/RA and sodium: ≤ 140 mg/RA [8]. Upper thresholds: total fat: > 10 g/RA, sugars: > 19 g/RA, sodium: > 360 mg/RA, and saturated fats: > 2 g/RA [8]. Tier 1 foods do not exceed lower thresholds for total fat, sugar and sodium. Tier 2 may exceed one or two lower thresholds but no upper thresholds. Tier 3 Vegetable and Fruit, and Grain Products: exceeds all lower threshold and no upper thresholds, or only one upper threshold. Tier 3 Milk and Alternatives, and Meat and Alternatives: exceeds all lower threshold and no upper thresholds, or only one upper threshold for total fat, sugars, or sodium. Tier 4 Vegetable and Fruit, and Grain Products: exceeds two upper thresholds. Tier 4 Milk and Alternatives, and Meat and Alternatives: exceeds upper thresholds for total fat, sugars, or sodium [8]. Tier 4 and “other foods” have no Canada’s food guide serving size recommendation. Compliers (Q1) are identified as the lowest 25% of the population with reported intakes from Tier 4 and “other” foods and beverages. Individuals categorized as “Intermediates” (Q2 and Q3) are those with intakes in the interquartile range. Non-compliers (Q4) represent 25% of the population with the highest intakes from Tier 4 and “other” foods. Physical activity in the 2015 CCHS was measured according to WHO guidelines (different from CCHS 2004) for children 6 to 17 years. Physical activity was defined as “activity that increases their heart rate and makes them feel out of breath some of the time” [11]. Physical activity in the 2015 CCHS measured the frequency of moderate to vigorous physical activity aimed to compare to WHO guidelines

Upon examination of nutrient intakes by quartiles (Table 4), the percentage of daily energy coming from dietary fibre, vitamin A, vitamin D, folate, vitamin B12, calcium, potassium, iron, and other nutrients decreased as energy intake from Tier 4 foods and “other foods” increased. The aforementioned reported nutrients were adjusted for misreporting, and intakes were significantly lower across quartiles (Q1-Q4) *p* < 0.001. In comparison, percent daily energy intake from saturated fat, and total fats increased significantly across quartiles. No difference across quartiles was seen for sodium, although reported intakes across all quartiles were high.

**Table 4:** Weighted analysis of nutrient intakes by compliers, intermediates, and non-compliers based on the percentage of energy consumed from Tier 4 and “other foods” and beverages among Canadian children adjusted for age, sex and misreporting status (under-reporter, plausible reporter, and over-reporters)

Nutrients	Compliers (Q1)	Intermediates (Q2)	Intermediates (Q3)	Non-compliers (Q4)	p-Trend
	< 10.9% Energy	10.9 -20.8% Energy	20.9 -32.9% Energy	> 32.9% Energy	
	Mean (SEM)	Mean (SEM)	Mean (SEM)	Mean (SEM)	
Energy (kcal/day)	1762 (32.3)	1978 (41.6)	1914 (32.6)	2040 (40.2)	< 0.0001
Fat (% Energy)	28.6 (0.4)	30.9 (0.4)	31.4 (0.4)	32.7 (0.4)	< 0.0001
Saturated fat (% Energy)	9.7 (0.2)	11.1 (0.2)	11.4 (0.2)	11.6 (0.2)	< 0.0001
Monounsaturated fat (% Energy)	10.5 (0.2)	10.9 (0.2)	11.1 (0.2)	11.7 (0.2)	< 0.0001
Polyunsaturated fat (% Energy)	5.7 (0.1)	6.0 (0.1)	6.0 (0.1)	6.6 (0.2)	< 0.0001
Carbohydrates (% Energy)	54.1 (0.5)	53.1 (0.5)	53.9 (0.4)	54.0 (0.4)	0.4137
Protein (% Energy)	17.3 (0.2)	16.0 (0.2)	14.7 (0.2)	13.0 (0.2)	< 0.0001
Alcohol (% Energy)	0 (0.0)	0 (0.0)	0 (0.0)	0.3 (0.1)	0.0676
Dietary fibre (g/1000 kcal)	10.2 (0.2)	9.0 (0.2)	8.4 (0.2)	7.1 (0.2)	< 0.0001
Vitamin A (RE/1000 kcal)	398.1 (26.0)	352.1 (11.1)	326.3 (10.3)	285.9 (9.0)	< 0.0001
Vitamin D (RE/1000 kcal)	3.6 (0.2)	3.1 (0.1)	2.8 (0.1)	2.6 (0.1)	< 0.0001
Thiamin (mg/1000 kcal)	1.0 (0.0)	0.9 (0.0)	0.9 (0.0)	0.7 (0.0)	< 0.0001
Niacin (mg/1000 kcal)	20.8 (0.3)	18.7 (0.3)	17.9 (0.2)	15.8 (0.3)	< 0.0001
Riboflavin (mg/1000 kcal)	1.1 (0.0)	1.0 (0.0)	1.0 (0.0)	0.9 (0.0)	< 0.0001
Vitamin B6 (ug/1000 kcal)	0.9 (0.0)	0.8 (0.0)	0.7 (0.0)	0.7 (0.0)	< 0.0001
Folate (ug/1000 kcal)	112.2 (2.9)	103.2 (2.3)	90.7 (1.8)	74.3 (1.7)	< 0.0001
Vitamin B12 (ug/1000 kcal)	2.6 (0.3)	2.2 (0.1)	1.9 (0.1)	1.7 (0.1)	< 0.0001
Vitamin C (mg/1000 kcal)	68.7 (3.0)	67.4 (2.5)	67.2 (2.6)	56.2 (2.2)	0.0064
Calcium (mg/1000 kcal)	560.6 (10.9)	552.4 (9.0)	495.2 (9.4)	430.7 (10.5)	< 0.0001
Phosphorus (mg/1000 kcal)	772.9 (10.1)	714.1 (7.2)	659.4 (7.1)	598.6 (8.9)	< 0.0001
Potassium (mg/ 1000 kcal)	1528.4 (20.4)	1387.6 (15.7)	1270.6 (16.3)	1094.1 (14.3)	< 0.0001
Sodium (mg/ 1000 kcal)	1431.8 (23.5)	1423.0 (18.1)	1459.1 (20.8)	1375.5 (20.9)	0.0509
Magnesium (mg/ 1000 kcal)	165.2 (2.3)	149.8 (1.5)	138.6 (1.7)	120.3 (1.7)	< 0.0001
Iron (mg/ 1000 kcal)	7.2 (0.1)	6.8 (0.1)	6.8 (0.1)	6.1 (0.1)	< 0.0001
Zinc (mg/ 1000 kcal)	5.7 (0.1)	5.3 (0.1)	4.8 (0.1)	4.2 (0.1)	< 0.0001

Results are presented as mean (SEM: standard error of the mean). Quartiles are based on the percentage of energy reported for all Tier 4 foods and “other” foods and beverages classified according to lower and upper thresholds for nutrients of public health concern: sodium, saturated fats, total fats and sugars. Lower thresholds: total fat: ≤ 3 g/RA, sugars: ≤ 6 g/RA and sodium: ≤ 140 mg/RA [8]. Upper thresholds: total fat: > 10 g/RA, sugars: > 19 g/RA, sodium: > 360 mg/RA, and saturated fats: > 2 g/RA [8]. Tier 1 foods do not exceed lower thresholds for total fat, sugar and sodium. Tier 2 may exceed one or two lower thresholds but no upper thresholds. Tier 3 Vegetable and Fruit, and Grain Products: exceeds all lower threshold and no upper thresholds, or only one upper threshold. Tier 3 Milk and Alternatives, and Meat and Alternatives: exceeds all lower threshold and no upper thresholds, or only one upper threshold for total fat, sugars, or sodium. Tier 4 Vegetable and Fruit, and Grain Products: exceeds two upper thresholds. Tier 4 Milk and Alternatives, and Meat and Alternatives: exceeds upper thresholds for total fat, sugars, or sodium [8]. Tier 4 and “other foods” have no Canada’s food guide serving size recommendation. Compliers (Q1) are identified as the lowest 25% of the population with reported intakes from Tier 4 and “other” foods and beverages. Individuals categorized as “Intermediates” (Q2 and Q3) are those with intakes in the interquartile range. Non-compliers (Q4) represent 25% of the population with the highest intakes from Tier 4 and “other” foods

## Discussion

This study is the first study to our knowledge to apply HCST to examine the quality and quantity of the food choices of children ≤18 years using the CCHS 2015, nutrition data. This evaluation provides relevant dietary intake data for children and adolescents at a national level, across quartiles of diet quality, identifying the pressing need to improve food choices.

Our findings, like other studies in the literature, have shown that minimal changes have occurred with Vegetable and Fruit consumption over the years. Analysis by Jessri et al., of the 2004 CCHS data indicated children 4–18 years were not meeting the minimum recommendations outlined by EWCFG 2007 [13, 17]. Similarly, according to the larger 2016 annual CCHS examining population health, 70% of children 4–8 years, 62% (girls), and 68% (boys) 9–13 years reported consuming less than the recommended five servings of Vegetables and Fruits/day using a food frequency questionnaire [19]. New food guidance suggests Vegetable and Fruit intake should reflect “half a plate” [7]. While no quantitative recommendations are in place yet, potential analysis according to new food guidelines may reflect poorer diets than expected, especially for older children as 62–70% of children were not meeting previous recommendations [9].

Packaged foods and beverages commonly marketed to children such as, chips, chocolate bars, soft drinks/sodas etc., are high in sugar, fats, and sodium and encourage poor dietary habits leading to NCDs [6, 20]. Many of these foods are captured in the “other foods” category according to HCST, this study provides a good indicator of children’s intake of food choices to limit. This study showed, on average that 21 - 24% of daily calories for children 4–18 years are consumed from the Tier 4 and “other foods” category, with the highest contributor being high fat and/or sugary foods. Interestingly, this study also showed changes in the quality of foods consumed, with poorer intakes seen during the transition to adolescence. Average energy intake from high calorie beverages was almost twice as high in children 9–13 years compared to 4–8 year olds, in both boys and girls. Additionally, a higher average intake from high fat and/or sugary foods, as well as saturated fats and/or trans fats was observed in the 9–13 year age groups. We observed no real difference in the number of servings of food according to EWCFG four food groups, but a difference in the nutritional quality of foods consumed during this time. It could be hypothesized that the change in the nutritional quality is a result of changes in preference of foods, in addition to more opportunities for children and adolescents to choose their own foods, and hence marketing to children will impact their choices.

Health Canada has taken action to limit consumption of packaged foods high in sugar, sodium, and fats with proposed new mandatory Front-of-Packaging labelling regulations [20, 21]. It is hoped that new mandatory regulations, if finalized, will improve dietary choices of Canadians, as minimal changes in the quality of diet have occurred during the intervening 11 years between the national nutrition surveys.

When comparing intakes to CCHS 2004, average daily calories coming from this “other foods” category decreased from 27 to 31% [17] to 21–24%. Caution needs to be used when interpreting changes between these two cycles of national nutrition data, as investigations by Statistics Canada revealed a higher level of under-reporting with CCHS nutrition 2015, in addition to an average 250 kcal decrease [14]. Possible explanations for these observed changes to intake may result from updates to the CNF over the intervening 11 years of the two survey cycles [14]. The CNF reflect changes and updates within the food industry/market. For example, compared to the last cycle of CCHS nutrition 2004, the estimated calories of “Italian dressing” declined three-fold [12, 14]. In addition to the updated CNF, updated design and pictures seen in the food module booklet may have impacted the estimation of amounts of foods consumed. Additionally, standardized food sizes have changed over the years which may have contributed to a larger percentage of under-reporting [11, 12, 14].

This study investigated energy intake according to quartiles of Tier 4 and “other foods”, with individuals in the lowest quartile categorized as compliers and in the highest quartile as non-compliers. Under-reporting ranged from 20 - 32% among children, BMI and physical activity remained relatively constant across all quartiles. Interestingly, individuals in lower quartiles did not have significantly better reported physical activity levels or lower measured BMI; this may indicate these measures are poor across all sub-groups. However, intakes of vitamin D, calcium, fiber, and most micronutrients decreased with poorer quality diets and daily saturated fats increased among non-compliers. This trend is not surprising as many nutrient poor foods are low in these nutrients. Intake of sodium (mg/1000 kcal) was high across all quartiles 1375.5 (20.9) - 1459.1 (20.8) indicating high sodium intake across all Canadians and thus remains a target for Canada’s Sodium Reduction Strategy and continued efforts are needed to reach sodium reduction targets [10, 22].

This study is the first to our knowledge to provide up- to- date dietary information using the CCHS 2015, and Health Canada’s Tier system, a nutrient profiling model, to examine the quality and quantity of the food choices of Canadian children and adolescents. Importantly the CCHS 2015 interviewed individuals for the 24-h dietary recall on all days of the week, and throughout the entire year to account for day-to-day and seasonal variations in reporting. This study is not without its limitations. Findings in this study used the 2014 HCST, which categorizes foods into Tiers according to thresholds for four nutrients of concern (i.e., sodium, sugar, saturated fats, and total fats). Future work could update or modify the 2014 HCST to include guidelines for recommended nutrients such as calcium, Vitamin D, iron, fibre and others. Additionally, updating the 2014 HCST is needed to reflect recent changes in Canada’s new Food Guide which includes quantitative recommendations such as half a plate for Vegetables and Fruits, and a quarter of a plate for each of Protein and Grain Products [7]. Examination of current dietary intakes against Canada’s new food guide may reveal Canadians are eating poorer than expected, however this remains an area where further research is needed [7]. Alternatively, future research could combine HCST analysis with a broader diet quality score to help make further comparisons across DRI age-sex groups.

## Conclusions

The majority of the dietary intake of Tier 1 foods are from Vegetables and Fruit consumption, however the quantity consumed compared to Canada’s dietary recommendations is low for children 9 years and older. The nutritional quality of foods consumed by children and adolescents were mainly from Tier 3 followed by Tier 2 foods. Within all age groups, as intake from Tier 4 and “other foods” increased, the nutritional quality of the diet decreased. This data provides support to limit the marketing of unhealthy processed and packaged foods to children to reduce their consumption. Furthermore, Front-of-Package labeling will help parents and children identify foods poor in nutritional quality. Future studies are needed to analyze the consumption patterns of children once an updated Tier system is introduced that categorizes food choices and diet with the new food guide.

## Data Availability

The data that supports the findings of this study are available from **Statistics Canada Toronto Research Data Center,** but restrictions apply to the availability of these data, which were used under the license for the current study, and so are not publicly available. Data are however available from the authors upon a reasonable request and with permission of **Statistics Canada Toronto Research Data Centre**.

## Abbreviations

AMPM: Automated multi-pass method
BMI: Body mass index
CCHS: Canadian Community Health Survey
CFG: Canada’s food guide
CNF: Canadian Nutrient File
DV: Daily value
EER: Estimated energy requirement
EI: Energy intake
EWCFG: Eating well with Canada’s food guide
GBD: Global burden of disease report
HCST: Health Canada Surveillance Tool, Tier System
IOM: Institute of Medicine
NCDs: Non-communicable diseases
Q: Quartile
RA: Reference amount
RDC: Research Data Centre
TEE: Total energy expenditure
USDA: United States Department for Agriculture
